# MicroRNAs for the Diagnosis and Management of Malignant Pleural Mesothelioma: A Literature Review

**DOI:** 10.3389/fonc.2018.00650

**Published:** 2018-12-21

**Authors:** Giuseppe Lo Russo, Anna Tessari, Marina Capece, Giulia Galli, Filippo de Braud, Marina Chiara Garassino, Dario Palmieri

**Affiliations:** ^1^Department of Medical Oncology, Fondazione IRCCS Istituto Nazionale dei Tumori, Milan, Italy; ^2^Department of Cancer Biology and Genetics, the Ohio State University, Columbus, OH, United States; ^3^Department of Oncology and Hemato-Oncology, Università degli Studi di Milano, Milan, Italy

**Keywords:** miRNAs, malignant pleural mesothelioma biomarkers, diagnosis, prognosis, therapy

## Abstract

Malignant pleural mesothelioma (MPM) is a rare and aggressive tumor with a variable incidence among different countries. Occupational asbestos exposure is the most important etiological factor and a very long latency period is widely reported. In the early phase of the disease, clinical signs are absent or not specific. For this reason, the diagnosis is frequently achieved only in the advanced stages. The histopathological diagnosis *per se* is also very complex, and no known factor can predict the prognosis with certainty. Nonetheless, current survival rates remain very low, despite the use of standard treatments, which include surgery, chemotherapy and radiotherapy. The identification of new prognostic and/or diagnostic biomarkers, and the discovery of therapeutic targets is a priority and could lead to a real significant impact on the management of malignant pleural mesothelioma. In this scenario, the role of microRNAs is becoming increasingly relevant, with the promise of a quick translation in the current clinical practice. Despite the relative novelty of this field, the number of works and candidate microRNAs that are present in literature is striking. Unfortunately, to date the microRNAs with the most clinical relevance for MPM are still matter of debate, probably due to the variety of approaches, techniques, and collected samples. Although specific microRNAs (e.g., let-7, miR-15 and miR-16, miR-21, miR-34a, and the miR-200 family) have been reported several times from different groups, the heterogeneity of published data reinforces the need of more comprehensive and unified studies on this topic. In this review we collect and discuss the studies focused on the involvement of microRNAs in different aspects of MPM, from their biological role in tumorigenesis and progression, to their possible application as diagnostic, prognostic and predictive biomarkers. Lastly, we examine their potential value as for the design of therapeutic approaches that could benefit MPM patients.

## Introduction

Malignant pleural mesothelioma (MPM) is a rare form of cancer originating from mesothelial cells of the pleura and generally characterized by a poor prognosis. The highest incidence is reported in the sixth and seventh decade of life. This tumor is more common in males than in females. The overall survival (OS) is about 10 months for advanced disease, with a 5% 5-years survival rate. Globally, MPM is responsible for 4% of cancer deaths in both men and women (1, 2).

A cause-effect relationship to asbestos exposure is widely reported, with symptoms that become often evident after a long latency period. Because of this, a peak in the incidence of MPM is awaited around 2030, due to the high exposure to asbestos in past years in several countries (3). Other recognized risk factors are radiation exposure, genetic mutations and the exposition to Simian Virus 40 (4).

The most common subtype of MPM is the epithelioid subtype (55–65%), followed by biphasic (15–20%), and sarcomatoid (10–15%) forms (5). The median OS is strongly influenced by histology, with lower survival rates for sarcomatoid patients in comparison with epithelioid ones (6).

The diagnosis of MPM displays several layers of complexity. Firstly, symptoms and imaging analyses are not disease-specific. Moreover, the cytological examination of pleural fluid is frequently possible only in advanced stages, and leads to specific diagnosis in a minority of cases (7–9). Pleural biopsy is the gold-standard diagnostic tool, but it can be affected by adverse events like pleural bleeding, infections, empyema, and pneumothorax (10). The histopathological diagnosis *per se* is also very difficult because of the lack of immunohistochemical markers with high specificity, and it requires the presence of a particular combination of positive/negative markers evaluated by an expert pathologist, especially when the goal is the differential diagnosis of MPM subtypes (11, 12).

In recent decades, the identification of specific molecular targets and genetic alterations has radically changed the therapeutic paradigms for different types of cancer, but has not significantly affected the natural history of MPM. In MPM both the role of surgery and radiotherapy is controversial. Since 2003, the only treatment that has clearly shown an improvement of patients survival is the standard chemotherapy with platinum and pemetrexed (13, 14). Both the use of different targeted biological agents and immunotherapy with anti-CTLA4 did not show a relevant efficacy even if many other checkpoint inhibitors (anti-PD1/PD-L1) and new generation compounds are still being investigated (13, 14).

In this difficult context, new prognostic or predictive biomarkers, new diagnostic approaches, and therapeutic targets are needed and could have a significant impact in the clinical management of MPM. Among these, the role of microRNAs (miRNAs) is becoming increasingly relevant. MiRNAs are small non-coding RNAs of about 22 nucleotides, playing an important role in post-transcriptional regulation of the expression of all human genes. For this reason, miRNAs affect any cellular process, including cell proliferation, apoptosis, and migration (15, 16). Altered expression of specific miRNAs has been associated with multiple human diseases, including cancer. Notably, differential expression of miRNAs in healthy vs. cancer tissues of different origins has been described, confirming the causal role of miRNAs in multiple aspects of cancer pathogenesis, ranging from tumor establishment to progression, metastasis and resistance to therapies (15, 16). Therefore, specific miRNA expression signatures may correlate with different patient prognosis or response to therapeutic approaches. Nonetheless, miRNAs can be quantified in multiple biological fluids, such as blood, cerebrospinal fluid, urine, and saliva. Also in these cases, specific signatures for cancer vs. normal patients have been identified. Altogether, these features make miRNAs ideal candidates as prognostic, predictive and diagnostic biomarkers (16–18).

Finally, the modulation of miRNA expression, by inhibiting those with oncogenic properties or rescuing the tumor-suppressive ones, represents a new exciting topic in the development of novel anti-cancer therapies (15–18).

The aim of this review is to describe the role of miRNAs in MPM with a specific focus on the research state of the art and on the potential translation in the current clinical practice.

## Biological Roles of miRNAs in MPM

### MiRNA Signatures

The first observations regarding the biological role of miRNAs in MPM date back to 2009. Using miRNA microarray technique (Agilent human miRNAs V2) and normal human pericardium tissues as controls, Guled et al. demonstrated a different miRNA expression profile between MPM and non-cancer tissues. By analyzing 17 MPM samples and testing 723 miRNAs, they showed a lower expression of let-7e, miR-7-1, miR-9, miR-34a, miR-144, miR-203, miR-340, miR-423, miR-582, and a higher expression of let-7b, miR-30b, miR-32, miR-195, miR-345, miR-483-3p, miR-584, miR-595, miR-615-3p, miR-1228 in neoplastic tissues compared with normal ones. The majority of these miRNAs were either located in chromosomal areas generally known as aberrant in MPM, or were targeting well-described genes involved in MPM tumorigenesis. Over-expressed miR-30b, miR-32, miR-483-3p, miR-584, and miR-885-3p target tumor-suppressor genes such as *CDKN2A, RB1*, and *NF2*. Conversely, down-regulated miR-9, miR-7-1, and miR-203 target *EGF, HGF, JUN*, and *PDGFA* oncogenes (19).

Balatti et al. evaluated miRNA expression profile in 5 human normal pleural mesothelial short-term cell cultures (HMCs) and 5 MPM tissue samples, with microarray approach. The comparative analysis of miRNA expression showed that miR-17-92 cluster and its paralogs, called miR-17-5p, 18a, 19b, 20a, 20b, 25, 92, 106a, 106b, were strongly up-regulated. Furthermore miR-7, miR-182, miR-214, and miR-497 were showed to be dysregulated in MPM. Intriguingly, these miRNAs were predicted (and later partially confirmed) to target genes involved in the regulation of cell cycle progression (20).

Ramirez-Salazar et al analyzed, using PCR Array (384 miRNAs), specimens obtained from 4 patients with pleural chronic inflammation, 5 patients with mesothelial hyperplasia, 5 patients with MPM and 4 normal controls with the aim to identify tumorigenesis-related miRNAs and their biological networks. MiR-101-3p and miR-494 were down-regulated in pleural chronic inflammation and mesothelial hyperplasia, respectively. In MPM tissues a reduction of miR-181a-5p, miR-101-3p, miR-145-5p, and miR-212-3p expression was observed. The down-regulation of these miRNAs resulted in increased levels of the mesenchymal transition-associated molecule FZDA, the transcription factor ETS1 and the signaling-activation molecule MAPK1, which have strong oncogenic functions. This suggested a possible association between pleural inflammation, hyperplasia and tumorigenesis (21).

Ak et al. also used PCR Array to compare miRNA signatures in 18 MPM and 6 non-cancer pleural tissue samples obtained from patients with benign asbestos-related pleural effusion. The study found 11 over-expressed miRNAs in MPM (let-7a, let-7d, miR-20a, miR-92a, miR-125a-5p, miR-152, miR-155, miR-193b, miR-320, miR-484, and miR-744). The authors further evaluated miRNA-mRNA interactions and found eight significant pathways targeted by miRNAs, including two related to NOTCH signaling. Compared to benign asbestos-related pleural effusion, *MET* was the most overexpressed gene in MPM (22).

In the study by Walter et al., NanoString technique was used to evaluate expression of 800 miRNAs from 24 formalin-fixed paraffin-embedded MPM samples. The principal aim was to define the impact of miRNA expression on the MDM2-P14/ARF (CDKN2A)-TP53 pathway, taking into account the differential immunohistochemical MDM2 expression (score 0 vs. score ≥1) in MPM tissues. Eleven miRNAs suppressing *CDKN2A* (miR-29a, miR-29b, miR-29c, miR-125a, miR-125b, let-7a, let-7c, let-7d, let-7e, let-7g, miR-340), 17 miRNAs inhibiting *TP53* (miR-29a, miR-29b, miR-29c, miR-125a, miR-125b let-7a, let-7c, let-7d, let-7e, let-7g miR-34a, miR-145, miR-185, miR-19b, miR-218, miR-22, miR-27b) and 18 miRNAs targeting *MDM2* (miR-29a, miR-29b, miR-29c, miR-125a, miR-125b miR-34a, miR-145, miR-185, miR-140, miR-223, miR-23b, miR-142, miR-191, miR-331, miR-605, miR-548d, miR-374b, miR-383) were down-regulated in MDM2-expressing MPM. Since MDM2 and CDKN2A expression regulates *TP53* and may contribute to its inactivation, the authors concluded that TP53 may be suppressed by miRNAs depending on expression pattern, whereas the impact of miRNAs on CDKN2A and on MDM2 itself is mild (23).

Very recently, the same group published another paper based on the same case series. In this work the authors focused their attention on a small subset of miRNAs regulating key enzymes involved in DNA damage repair. Specifically, the pathways reported as mostly de-regulated were TP53 (let-7b-5p and miR-143-3p), PARP1 (miR-21-5p, miR-223-3p, miR-302d-3p), and RAD52 (miR-106a-5p, miR-106b-5p, miR-20a-5p) (24).

Lastly, Kim et al. investigated the global expression profile of miRNAs in distinct subpopulations of a MPM cell line (MS1). Their results showed that a subset of miRNAs is able to define the most aggressive cell subpopulations. ErbB-2 receptor tyrosine kinase signaling was the most involved pathway and *DDIT4* and *ROCK2* the most involved target genes. The specific miRNA signature defining aggressive subpopulations included over-expression of miR-3198-1, miR-3198-2, miR-4497, miR-138-1, miR-4304, miR-1281, miR-489, miR-4745, miR-301a, miR-3935, and down-regulation of miR-148b, miR-484, miR-584, miR-425, miR-197, miR-629, miR-183, miR-4485, miR-4443, and miR-1246 (25).

### Single miRNAs or miRNA Families

In regards to the role of single miRNAs or miRNA families in the pathogenesis of MPM, a wide literature has been published to date (Table 1) (26–42, 44–54).

**Table 1:** Biological roles of miRNAs in MPM.

**miRNA**	**Target genes/pathways**	**Cell function**	**References**
miR-1	*PIM1, TP53, BAX, P16/21, BCL2*	Apoptosis, proliferation, migration, invasion	(26, 27)
Let-7a/b	EphA1 signaling, *RAS, PARP*, Procaspases 3, Twist, b-catenin, *AKT, TP53*	EMT, apoptosis, proliferation, migration, invasion	(23, 24, 28–30)
miR-15a/16	*BCL-2, CCDN1, PD-L1, FGF* axis	Radio/chemo-sensitivity, apoptosis, proliferation, colony formation	(31–33)
miR-17-5p	*KCNMA1*	Migration	(34)
miR-21-5p	*PARP1, MSLN*	Proliferation	(24, 35)
miR-29c-5p	*TP53, DNMT1, DNMT3A*	Methylation, proliferation, colony formation, migration, invasion	(23, 24, 36)
miR-31	*PPP6C*	Radio/chemo-sensitivity, proliferation, colony formation, migration, invasion	(37)
miR-34a/b/c	*BCL-2, c-MET, CDKN2A, NF2, TP53*	Radio/chemo-sensitivity, apoptosis, methylation, proliferation, colony formation, migration, invasion	(38–43)
miR-126	*ACL, PDK, IRS1, HIF1α, EGFL7*	Autophagic flux, mitochondrial function, methylation, proliferation, migration, invasion	(44–46)
miR-137	*YBX1*	Proliferation, migration, invasion,	(47)
miR-145	*OCT4, ZEB1*	Proliferation, migration, invasion, colony formation,	(48)
miR-193a-3p	*MCL1, PD-L1*	Apoptosis, cell death, proliferation	(49)
miR-205	*ZEB1, ZEB2*	EMT, migration, invasion	(50)
miR-223	*PARP1, MDM2, TP53, JNK* signaling, *STMN1*	Tubulin acethylation, proliferation, migration	(23, 24, 51)
miR-302b	*MCL1*	Apoptosis, proliferation	(52)
miR-379, miR-411	*IL-18*	Chemo-sensitivity, proliferation, invasion	(53)

Pass et al. analyzed 12 MPM cell lines (9 neoplastic and 3 normal) and 142 MPM tumor samples. In MPM cell lines, the authors demonstrated that the over-expression of miR-29c decrease invasion, migration, proliferation, and colony formation. Furthermore, miR-29c over-expression mediated epigenetic mechanisms through the down-regulation of *DNMT1* and *DNMT3A* and the up-regulation of demethylating genes. The increased level of miR-29c in tumor samples was related with a better outcome after surgery. The authors hypothesized that miR-29c could play a tumor suppressive role in MPM and thus it may be a potential new therapeutic target (36).

Ivanov et al. reported that miR-31 plays a similar role in MPM. The loss of expression of miR-31 due to the deletion of miR-31 gene in chromosome 9p21.3 is a common aberration in the aggressive forms of MPM. The investigators demonstrated that miR-31 as well as miR-29c is able to block migration, proliferation, and invasion in MPM cell lines. Moreover, low miR-31 levels were associated with high levels of protein phosphatase 6 (PPP6C) which were related with radio and chemo-resistance. According to this study, the re-introduction of miR-31 in MPM patients could be another potential therapeutic approach (37).

One of the most studied miRNA family in MPM is represented by miR-34 (38–42).

Ghawanmeh et al. examined the effects of docetaxel and radiotherapy on MPM cell lines. In the M28K cells, radiotherapy induced miR-34a expression, cell cycle arrest and cell death (38). Kubo et al demonstrated that the epigenetic silencing of miR34b/c due to methylation, is crucial in the pathogenesis of MPM. In MPM cell lines the authors showed that physiologic miR-34b/c levels had anti-proliferative effects and that the forced over-expression of miR-34b/c had a pro-apoptotic effect (39).

The studies by Tanaka et al and by Maki et al confirmed these observations (40, 41). The first article reported that the down-regulation of miR-34a induced cell proliferation and invasion in MPM cells because of the consequent up-regulation of *c-MET* and *BCL-2* (40). The second paper demonstrated that high levels of miR-34b/c increased radiation-induced apoptosis and suggested that miR-34b/c could be used as radiosensitizing agent in MPM (41). Finally, Menges et al., proved *in vivo* that the inactivation of *CDKN2a* and *NF2* causes the development of MPM in mouse models. These tumors were characterized by TP53/miR-34a-dependent activation of c-MET, which correlated with high aggressiveness and presence of cancer stem cells (42). Some years after these studies, Yamamoto et al reported that miR-379/411 cluster directly target *IL-18* gene, whose over-expression was associated with drug resistance in MPM cell lines. *In vitro*, the introduction of miR-379 and miR-411 (with the consequent *IL-18* silencing) reduced invasiveness and increased chemosesnsitivity of MPM cells (53).

A large body of evidence has demonstrated the role of let-7 family in MPM pathogenesis. MiRNAs of this group (including more than 10 different members) have all a similar structure and have a huge number of functions and targets. Firstly, Khodayari et al. showed that EphrinA1 signaling inhibits MPM tumor growth by repressing RAS proto-oncogene family through let-7a. EphrinA1 is a specific ligand of the EphrinA2 receptor, which is over-expressed in most cancer cells, including MPM. In this work, the authors demonstrated that EphrinA1 binding to its receptor EphrinA2 suppresses MPM tumor growth through up-regulation of miR let-7a and the subsequent down-regulation of RAS proto-oncogenes family (28). Two years later the same group reported that the targeted delivery of miR let-7a, encapsulated in liposomal nanoparticles conjugated with EphrinA1, inhibits migration, proliferation, and tumor growth in MPM and NSCLC cell lines. This observation suggests a new possible therapeutic approach potentially useful especially in neoplasms overexpressing EphrinA2 receptor (29).

Sohn et al. focused their attention on another member of let-7 family. By transfecting let-7b synthetic mimic in H28, H2452, and MSTO-211H MPM cell lines treated with ursolic acid, they found that the up-regulation of let-7b was critically involved in ursolic acid induced apoptosis. The over-expression of let-7b increased the activity of ursolic acid leading to PARP and caspase 3 cleavage, and down-regulation of Twist, β-catenin and pAKT with a consequential sub-G1 cell accumulation and block of the epithelial to mesenchymal transition (EMT) (30).

In regards to the role of EMT in MPM pathogenesis, Fassina et al. have given an important contribution. They collected 109 MPM tissue samples (58 epithelioid, 25 biphasic, and 26 sarcomatoid) and showed that there is a switch in the expression from epithelial to mesenchymal markers going through the less aggressive epithelioid forms to the more aggressive biphasic and sarcomatoid histotypes. Moreover, overexpression of miR-205 in mesothelial (MeT-5A) and MPM cell lines (H2452 and MSTO-211H) caused a reduction in the expression of mesenchymal (*ZEB1* and *ZEB2*) and an increment of epithelial (*E-cadherin*) markers, which ultimately led to the inhibition of migration and invasion processes (50).

Using microarray transcriptional profiling and having normal pleural tissue as control, Xu et al. analyzed 25 MPM tissue samples and found lower miR-1 levels in neoplastic tissues. Accordingly, reduced proliferation and increased apoptosis of MPM cell lines (H513 and H2052) upon overexpression of miR-1 was observed. This suggests that miR-1 may act as tumor suppressor in MPM (26). These data were confirmed in the subsequent study by Amatya et al, where transfection of miR-1 and miR-214 mimic led to the down-regulation of the proto-oncogene PIM1 and to the inhibition of cell growth, invasion and migration (27).

Reid et al. firstly showed that miR-15/16 family is down-regulated in MPM tumor tissues and cell lines, and has a tumor suppressive role in MPM. In their experience, restoring miR-15/16 expression in MPM cell lines caused a reduction of cell proliferation and increased chemosensitivity. These phenomena were correlated with the down-regulation of specific genes such as *CCND1* and *BCL-2*. Using xenografts models, the authors described a relevant antitumoral activity for miR-16 mimic packaged in intravenously-administered “minicells” (31). Very recently, the same group demonstrated that miR-15a, miR-16, and also miR-193a-3p contribute to the regulation of programmed death ligand 1 (PD-L1) expression in MPM causing its down-regulation (32). Moreover, Schelch et al. observed a down-regulation of the fibroblast growth factor (FGF) axis after transfection with miR-15/16 mimics. The restoration of miR-15/16 caused growth reduction in MPM cell lines and the combined inhibition of BCL-2 (another miR-15/16 target) resulted in a synergistic activity (33).

Cioce et al. focused their attention on miR-145, showing that treatment of MPM cell lines with miR-145 agonists reduced the protumorigenic power of MPM cells and increased the sensibility to pemetrexed. These data were confirmed in animal models, in which restoration of miR-145 expression inhibited tumor growth. The authors found that miR-145 targeted *OCT4* reducing its level and the level of its transcriptional target *ZEB1*. Higher OCT4 levels were associated with resistance to chemotherapy and with tumor growth (48).

As described by Tomasetti et al., miR-126 displays an oncosuppressive role in MPM cells by targeting *IRS1*, leading to impaired mitochondrial function and cell growth. Moreover, they demonstrated that miR-126 initiates a metabolic program, which implies high autophagic flux and HIF1α stabilization, playing a protective role in MPM (44, 45). Andersen et al., analyzing MPM tumor tissues and non-neoplastic controls, showed that DNA-hypermethylation down-regulates miR-126 and its host gene *EGFL7* leading to a reduction in patients survival in MPM (46).

Birnie et al. identified reduced levels of miR-223 in MPM patient specimens. The authors demonstrated that miR-223 targets *STMN1*, a microtubule regulator that has been associated with MPM. Moreover, they displayed that STMN1 is also regulated by the JNK signaling. The overexpression of miR-223 in MPM cell lines reduced STMN1 levels with a consequential induction of tubulin acetylation and reduction of cell motility. Furthermore, miR-223 levels grew and STMN1 levels decreased after the re-expression of the JNK isoforms in JNK-null murine embryonic fibroblasts. Finally, STMN1 levels decreased after JNK signaling activation (51). As reported by Walter et al., miR-223 is also down-regulated in MPM expressing MDM2, a negative regulator of TP53 (23).

Williams et al. found a significant decrease of the levels of miR-192 and miR-193a-3p in MPM tumor samples compared with non-cancer tissues. In MPM cell lines, transfection of miR-193a-3p mimic induced apoptosis and reduced cell proliferation causing reduction of the expression of the anti-apoptotic protein MCL1, frequently over-expressed in MPM. These data were confirmed in xenograft models in which the use of minicells containing miR-193a-3p mimics reduced tumor growth and increased apoptosis (49). In MPM, MCL1 is also downregulated by miR-302b. Khodayari et al. demonstrated that the treatment with ephrinA1 leads to the over-expression of miR-302b, which inhibits MCL-1 expression with a consequential induction of apoptosis and reduction of cells proliferation (52).

Cheng et al demonstrated that KCa1.1, a calcium-activated potassium channel subunit alpha 1 encoded by the KCNMA1 gene, is a target of miR-17-5p. KCa1.1 was overexpressed in MPM cells lines and MPM tissues compared with non-cancer samples. Moreover, the transfection of MPM cells with miR-17-5p mimic reduced the expression of KCa1.1 and blocked MPM cells migration (34).

De Santi et al., using Next Generation Sequencing (NGS) and the “miR-CATCH” method (based on biotinylated DNA antisense oligonucleotides that capture mRNA), identified miR-21-5p as a functional regulator of mesothelin (MSLN) gene expression. Moreover, they demonstrated that treatment with miR-21-5p mimic may decrease the proliferation in MPM cell lines (35). In a different study, the same group suggested that miR-126, miR-15b, miR-145, miR-185, miR-197, and miR-299 play a role in the regulation of cell metabolism in MPM. Comparing miRNA expression profile of 96 MPM patients with 10 non-cancer controls they found a significant down-regulation of these miRNAs in MPM. The top five pathways significantly affected by the deregulated miRNAs were: fatty acid biosynthesis, focal adhesion, MAPK, P53, and WNT signaling pathway (54).

Finally, few months before the submission of our review, Johnson et al., using MPM cell lines,127 MPM tissue samples (3 different cohorts), and 23 pleural or pericardium tissue controls, showed that miR-137 can exhibit a tumor-suppressive role in MPM by targeting Y-box binding protein 1 (YBX1). YBX1 knockdown significantly reduces tumor growth, migration, and invasion of MPM cells (47).

## Tissue Expression of miRNAs as Prognostic and Diagnostic Biomarkers in MPM

The previously cited paper by Pass et al is the first work proposing microRNAs as potential prognostic biomarkers in MPM. Using a custom miRNA expression analysis platform, a training set of 44 and a test set of 98 MPM tumor samples were analyzed. In both training and test sets, higher levels of tissue miR-29c was shown to be an independent prognostic factor for higher OS and time to progression (TTP) after surgery (36). Using qRT-PCR, Matsumoto et al measured miR-31 expression in 25 tissue samples obtained from MPM patients and in 20 tissues of patients with reactive mesothelial proliferations (RMPs). They displayed that the expression of miR-31 was reduced in MPM compared with RMPs. However, the up-regulation of miR-31 was associated with the presence of sarcomatoid component and with worse prognosis in patients affected by this histological tumor sub-type (55). Likewise, in the study by Busacca et al., low cancer tissue levels of miR-17-5p and miR-30c were associated with better OS in sarcomatoid MPM patients. Moreover, miR-30c was described as differentially expressed in the three MPM histotypes (56). Lastly, in the study by Fassina et al., the tissue levels of miR-205 were reported as lower in the more aggressive biphasic and sarcomatoid MPM histotypes and higher in the epithelioid forms characterized by better prognosis (50). Obviously, all these studies also suggest a role of miR-31, miR-17-5p, miR-30c, and miR-205 in the differential histopathological diagnosis of MPM.

The prognostic role of miRNAs in MPM has been demonstrated by different other studies (Tables 2–4) (24, 47, 54, 73–75, 78, 79).

**Table 2:** miRNAs associated with oncogenic mechanisms in MPM.

**References**	**Samples**								**Assay**				**miRNA**
	**MPM tissue**	**Cytological component of pleural effusion**	**Serum**	**Plasma**	**Cellular fraction of peripheral blood**	**Other tumor**	**Non-cancer related thoracic disease**	**Normal control**	**Microarray**	**PCR array**	**qRT-PCR**	**ISH**
Guled et al. (19)	17							1	X				Low: let-7e, miR-7-1,-9,-34a,-144,-203,-340,-423,-582. High: let-7b, miR-30b,-32,-195,-345,-483-3p,-584,-595,-615-3p,-1228
Ramirez Salazar et al. (21)	5						9	4		X			Low: miR-18a-3p,-101-3p,-145-5p,-181a-5p,-212-3p,-501-3p,-517b-3p,-596,-627,-671-3p,-766-3p. High: let-7-g-5p, miR-18a-5p,-34a-3p,-135b-5p,-196b-5p,-302b-3p,-622
Xu et al. (26)	25							6	X		X		Low: miR-1,-206,-483-5p. High expression: miR-155
Reid et al. (31)	60							23			X		Low: miR-16,-15a,-15b,-195
Cioce et al. (48)	29 + 6 + 36						12	14 + 36	X		X		Low: miR-145
Birnie et al. (51)	17	26					10	6			X		Low: miR-223

**Table 3:** miRNAs associated with MPM diagnosis.

**References**	**Samples**								**Assay**				**miRNA**	**Specificity**	**Sensitivity**
	**MPM tissue**	**Cytological component of pleural effusion**	**Serum**	**Plasma**	**Cellular fraction of peripheral blood**	**Other tumor**	**Non-cancer related thoracic disease**	**Normal control**	**Microarray**	**PCR array**	**qRT-PCR**	**ISH**		
Ak et al. (22)	18						6			X			let-7a, let-7d, miR-20a,-92a,-125a-5p,-152,-155,-193b,-320,-484,-744	≥80%	≥80%
Benjamin et al. (57)	7 + 32 + 16 + 14					97 + 113 + 23 + 49			X		X		Low: miR-200c,-192. High: mi-193a-3p	94%	100%
Gee et al. (58)	15 + 100					10 + 32			X		X		Low: miR-141,-200b,-200c,-203,-205,-429	>80%	>80%
Andersen et al. (59)	10 + 52							5 + 14		X	X		Low: mir-126,-143,-145,-652	>80%	>80%
Santarelli et al. (60)	10 + 27							5 + 27		X	X		Low: mir-126	
Cappellesso et al. (61)	51	29					40 + 24				X		Low: miR-126. High expression: miR-19a,-19b,-21,-25	87%	86%
Cappellesso et al. (62)	41	26				40 + 26					X		High: miR130a	67%	77%
Santarelli et al. (60)			44				196	50			X		Low: mir-126	
Santarelli et al. (63)			45 + 18			42	99 +50	44 +20			X		Low: mir-126	
Gayosso-Gómez et al. (64)			11			36		45	High throughput sequencing				High: miR-92b-5p,-96-5p,-185-5p,-409-5p,-1271-5p,-1292-5p,-4791	
Kirschner et al. (65)	18		30	5 + 15			10	3 + 14 +7	X		X		High: miR-625-3p	≥80%	≥80%
Bononi et al. (66)			10 + 20				10 + 15	10 + 14	X		X		High: miR-1281,-32-3p,-197-3p	
Weber et al. (67)				21 + 22			21 + 44			X	X		Low: miR-132-3p	61%	86%
Matboli et al. (68)			60				20	20			X		High: miR-20a,-548a-3p	87.5%	100%
Cavalleri et al. (69)				23 (exosomes)			19		Open Array		X		Low: miR-30e-3p,-103a-3p	80%	95.5%
Weber et al. (70)					23 + 23		17 + 17	25	X		X		Low: miR-103a-3p	76%	78%
Weber et al. (71)					43		52				X		Low: miR-103a-3p	81%	95%
Muraoka et al. (72)			48				21	41			Digital methylation PCR		High methylation: miR-34b/c	

**Table 4:** miRNAs associated with MPM prognosis.

**References**	**Samples**								**Assay**				**miRNA**
	**MPM tissue**	**Cytological component of pleural effusion**	**Serum**	**Plasma**	**Cellular fraction of peripheral blood**	**Other tumor**	**Non-cancer related thoracic disease**	**Normal control**	**Microarray**	**PCR array**	**qRT-PCR**	**ISH**
Mairinger et al. (24)	24								X				miR-1, 146a-5p,-335-5p,-378a-3p,-451a,-566,-1246
Pass et al. (36)	44+98								X		X		miR-29c-5p
Fassina et al. (50)	74										X	X	miR-205
De Santi et al. (54)	52 + 16								X		X		let-7c-5p, miR-151a-5p
Johnson et al. (47)	115 + 12							23			X		miR-137
Matsumoto et al. (55)	25										X		miR-31
Busacca et al. (56)	24								X		X		miR-17-5p,-30c
Kirschner et al. (73)	64 + 43								X		X		miR-21-5p,-23a-3p,-30e-5p,-31-5p,-221-3p,-222-3p
Truini et al. (74)	27 + 30							4	X		X		let-7c, miR-99a,-125b
Mozzoni et al. (75)	32			32			15 + 14		X		X		miR-16, miR-17, miR-126, miR-486
Tomasetti et al. (76)			45			20		56			X		mir-126
Lamberti et al. (77)			14				10			X	X		miR-191, miR-223. High: miR-25,-26b,-29a,-101,-335,-433,-516

In particular: high miR-137 (47) and miR-1, miR-335-5p, miR-566 (24) tissue levels have been correlated to poor prognosis, while the high tissue expression of miR-146a-5p, miR-378a-3p, miR-451a, miR-1246 (24), and of miR-16, miR-486 (75) was positively related with a better outcome.

Kirschner et al. identified a 6-miRNA signature (miR-Score) predictive of higher OS in patients with MPM who underwent extrapleural pneumonectomy with or without induction chemotherapy, including miR-21-5p, miR-23a-3p, miR-30e-5p, miR-221-3p, miR-222-3p, and miR-31-5p.14 (73). The 6-miR-Score has been subsequently modified first into a 2-miR-Score for use in diagnostic chemo-naïve specimens (78), than in a combined 2-miRNA-clinical score prognostic in both chemo-naïve and treated patients (79). The 2-miR-Score includes miR-221-3p.

De Santi et al. also identified a 2-miRNA prognostic signature. In the 52 MPM tissue samples analyzed, higher levels of let-7c-5p and miR-151a-5p were associated with poorer OS. These data were confirmed in a second cohort of 16 fresh/ frozen MPM (54).

Finally, using a microarray platform and 27 tissue samples obtained from un-resected MPM patients, Truini et al. performed a whole miRNA profiling and selected mir-99a, let-7c, and miR-125b as potential prognostic miRNAs. The signature was tested on public miRNA sequencing data from 72 MPM patients with available OS and validated by RT-qPCR in an independent set of 30 MPM patients. The authors found that the down-regulation of the miR-99a/let-7/miR-125b miRNA cluster was able to predict poor outcome in unresected MPM (74).

In regards to the potential role of miRNA tissue expression as diagnostic biomarkers, a large number of studies have been published (Tables 2–4) (19–22, 26, 27, 48, 51, 57–60).

Guled et al. identified specific miRNA profiles in tumor and non-cancer tissues, associated with specific histopathological subtypes (19).

Using microarrays, Benjamin et al. identified a different miRNA tissue expression profile between different types of cancer and MPM. MiR-193-3p levels were higher in MPM, while miR-192 and miR-200c levels were higher in lung primary adenocarcinomas and pleural metastases. In a blinded validation set of 68 samples the assay had a specificity of 94% and a sensitivity of 100% (57).

With the aim to identify a miRNA signature able to distinguish between MPM and lung adenocarcinoma, Gee et al., using microarrays, analyzed 15 MPM and 10 lung adenocarcinoma tissue samples. The results were validated by RT-qPCR in 32 lung adenocarcinoma and 100 MPM samples, respectively. MiR-141, miR-200b, miR-200c, miR-203, miR-205, and miR-429 were down-expressed in MPM and resulted able to discriminate MPM from lung adenocarcinoma (58).

Santarelli et al. tested fresh or frozen biopsies of MPM for the expression of 88 miRNAs and compared the results with non-cancer tissue controls. They found that miR-126 was significantly down-regulated in neoplastic tissues (60).

Ak et al. showed a significant up-regulation of multiple miRNAs in MPM tissue samples, and demonstrated that let-7a, miR-125a-5p, miR-320, and miR-484, were able to discriminate MPM from benign asbestos related diseases (22).

Andersen et al. demonstrated a potential diagnostic value of miR-126, miR-143, miR-145, and miR-652 in MPM. They screened with a (RT-qPCR)-based platform the expression of 742 miRNAs in 5 MPM tissue samples of patients previously treated with chemotherapy, 5 preoperative diagnostic biopsies of patients with MPM and 5 non-neoplastic pleura samples obtained from patients with MPM diagnosis after chemotherapy treatment. The author showed that miR-126, miR-143, miR-145, and miR-652 levels were significantly reduced in MPM samples compared with non-cancer pleural tissues. The results were validated by RT-qPCR in a cohort of 40 independent MPMs. However, we have to take into account that chemotherapy may induce changes in the miRNA expression both in neoplastic and non-cancer tissues and this is the biggest limitation of this study (59).

## Expression of miRNAs in Pleural Effusion

The detection and the quantification of miRNAs in pleural effusion cells have a great potential for the identification of new minimally-invasive diagnostic biomarker (Tables 2–4).

Firstly Birnie et al., using RT-qPCR, showed that in comparison with non-cancer specimens, miR-223 levels were significantly reduced both in the cellular component of the pleural effusion of MPM patients and in MPM tissue samples. They compared 6 non-neoplastic with 17 neoplastic tissue samples and 10 pleural effusion specimens obtained from patients with benign pleural diseases with 26 coming from MPM patients (51).

In a first work from Cappellesso et al., analyzed the expression of 15 selected miRNAs in one normal mesothelial (MeT-5A) and two neoplastic (H2052 and H28) MPM cell lines using RT-qPCR. MiRNAs were also tested in 51 MPM and 40 non-neoplastic pleural tissue samples, and validated in 29 neoplastic and 24 non-neoplastic pleural effusion cytologic specimens. Compared with non-neoplastic controls, miR-19a, miR-19b, and miR-21 were over-expressed, and miR-126 was under-expressed in tumor samples. The authors concluded that miRNAs were detectable in the cytologic component of MPM pleural effusion, and especially the combination of miR-21 and miR-126 could be useful in the MPM diagnosis, reporting 86% sensitivity and 87% specificity (61).

In a second study, the authors investigated the significance of miRNAs in the differential diagnosis between lung adenocarcinoma and MPM pleural effusion. A pool of selected miRNAs was analyzed by RT-qPCR in 41 vs. 40 tissue samples and in 26 vs. 27 cytological pleural effusion specimens obtained from MPM and lung adenocarcinoma patients, respectively. The authors showed that miR-130a, miR-141, miR-193a, miR-205, miR-375, and miR-675 were differentially expressed in the two tumors, but only miR-130a was significantly overexpressed in MPM compared with lung adenocarcinoma. The sensitivity and specificity of miR-130a quantification in the differential diagnosis were 77 and 67%, respectively (62).

## MiRNA Expression in Serum, Plasma and Cellular Fraction of Blood

It has been demonstrated that miRNAs are secreted in blood and serum, where they can be found both as soluble/protein associated, or included in lipid vesicles such as exosomes. For mechanisms that are still not completely known, cancer cells release a higher amount of circulating miRNAs, whose composition reflects the one present in the secreting cells. For this reason, the detection and quantification of circulating cancer-derived miRNAs might represent an extremely valuable tool for the management of different tumor types, including MPM (Tables 2–4) (15–18).

Using RT-qPCR Santarelli et al. compared the serum levels of miR-126 obtained from 50 healthy controls, 196 asbestos-exposed, and 44 MPM patients. The authors reported that cut-off values of miR-126 could significantly differentiate asbestos-exposed subjects from healthy controls and from MPM group. Moreover, the association between low levels of miR-126 and high levels of the specific MPM markers such as soluble mesothelin-related peptides (SMRPs) was able to identify subjects with high risk to develop MPM (60).

One year later the same group published another paper with the aim to investigate the accuracy and precision of circulating miR-126 quantification as clinical biomarker. The authors evaluated miR-126 serum levels in 56 healthy subjects, 20 non-small-cell lung cancer and 45 MPM patients, using both absolute and relative qRT-PCR methods. MiR-126 serum levels were reduced in both tumor types and associated with worse prognosis in MPM. Moreover, the quantification of miR-126 differentiated MPM from both normal controls and non-small-cell lung cancer, but it was not able to discriminate healthy controls from non-small-cell lung cancer (76).

In a third paper, Santarelli et al. combined the quantification of circulating miR-126 with SMRPs and methylated thrombomodulin promoter (Met-TM) serum determination. A total of 44 healthy controls, 99 asbestos-exposed, and 44 MPM patients were evaluated. The combination of high SMRP and Met-TM levels with low levels of miR-16 was evaluated as the best method to distinguish MPM from the other two groups. Moreover, in non-neoplastic subjects, the association between high SMRP levels and high Met-TM or low miR-16 levels, increased significantly the MPM risk. These data were confirmed in a validation cohort of 20 healthy controls, 50 asbestos-exposed subjects, 18 MPM, and 42 lung cancer patients (63).

With the aim to discriminate between lung adenocarcinoma and MPM diagnosis, Gayosso-Gómez et al. studied miRNA profile of serum samples obtained from healthy subjects (*N* = 45), lung adenocarcinoma (*N* = 36), and MPM patients (*N* = 11). Among known miRNAs, in comparison with normal controls, 12 miRNAs were overexpressed in lung adenocarcinoma, and 7 in MPM. Three of these were up-regulated only in MPM (miR-92b-5p, miR-409-5, and miR-1292). These differences could be very useful in the differential diagnosis process (64).

The potential role of increased circulating levels of miR-625-3p as biomarker for MPM has been showed by Kirschner et al. Firstly, using microarray analyses, the authors tested 90 miRNAs previously reported as associated with MPM in plasma samples of a cohort of 5 MPM patients and 3 healthy subjects, and found 15 miRNAs with higher levels in MPM patients compared with controls. Using qRT-PCR, the results were validated in a second cohort of plasma samples obtained from 14 non-neoplastic subjects and 15 MPM patients, and in a third cohort of serum samples obtained from 10 patients with asbestosis and 30 with MPM. In the three cohorts, only the high concentration of miR-625-3p was always able to discriminate between MPM and non-MPM patients. The up-regulation of miR-625-3p in MPM was also confirmed in a forth cohort of tissue samples (6 normal pericardial and 18 MPM tissues) (65).

Lamberti et al. collected serum samples from 14 patients with MPM and 10 patients affected by non-cancer-related pleural effusions, and performed a miRNA profiling using low-density microarray Real Time PCR system. They found two miRNAs exclusively expressed (miR-516 and miR-29a), two miRNAs down-regulated (miR-223 and miR-191), and five miRNAs up-regulated (miR-335, miR-25, miR-26b, miR-101, and miR-433) in MPM samples compared with non-cancer controls. Patients with MPM were divided into two miRNA serum signature groups: signature A (patients with more than 3/9 up-regulated miRNAs or 3/9 up-regulated miRNAs and miR-516 unchanged or not recordable) and signature B (patients with at least 3/9 down-regulated or unchanged miRNAs and/or miR-29a down-regulated). MPM patients with signature B had longer OS in comparison with patients with signature A (17 vs. 7 months). The authors also reported that signature A was associated with sarcomatoid or biphasic histology (5/5 patients), nevertheless they did not report the statistical value. However, this study displayed the limitations of a low number of patients enrolled and the use of patients with pleural effusions as controls instead of healthy subjects (77).

In the study by Bononi et al., serum circulating miRNAs from 10 healthy subjects, 10 asbestos-exposed and 10 MPM patients were analyzed with microarray and validated by qRT-PCR in a second cohort of 14 healthy controls, 15 asbestos-exposed, and 20 MPM patients (30 serum samples were previously used for microarray analysis). In MPM patients they found up-regulation of miR-1281 in comparison to both healthy subjects and asbestos-exposed patients, up-regulation of miR-32-3p and miR-197-3p in comparison only to asbestos-exposed patients and up-regulation of miR-32-3p, miR-197-3p, and miR-1281 in comparison only to healthy subjects. The authors concluded that these three miRNAs could be proposed as new MPM diagnostic biomarker (66).

Weber et al used TaqMan Low Density Array Human MicroRNA Cards to analyze 377 miRNAs in plasma samples obtained from 21 asbestos-exposed and 21 MPM patients. The results were validated in a second cohort of 44 asbestos-exposed and 22 MPM patients using RT-qPCR. The authors showed that miR-132-3p was significantly down-regulated in MPM and only this miRNA resulted able to well discriminate between MPM and asbestos-exposed patients with a reported specificity of 61% and sensitivity of 86%. MiR-126 was also reported as down-regulated in MPM but only in the validation cohort. The authors calculated a specificity of 86% and a sensitivity of 77% for the combined down-regulation of the two miRNAs used as diagnostic biomarker. Nevertheless, it is not clear why miR-126 was not reported as down-regulated also in the discovery cohort of this study (67).

Mozzoni et al. aimed to identify a miRNA signature helpful as diagnostic biomarker for asbestos-exposed and MPM patients. The authors collected tissue and plasma samples from patients affected by MPM (32 cases), asbestosis (14 cases), and other non-cancer pulmonary diseases (15 cases, used as controls). MiR-16, miR-17, miR-126, and miR-486 levels were quantified in plasma and tissues using qRT-PCR and all resulted decreased both in patients with asbestosis and in MPM, compared to controls. The levels of miR-486, miR-17, and miR-16 were significantly correlated in MPM tissue and plasma samples. Moreover, the tissue expression of miR-16 and miR-486 and the plasma levels of miRNA-16 were positively related with OS (75).

Lastly, Matboli et al., in a very recent paper, showed that the quantification of serum miR-548a-3p and miR-20a levels is a promising new diagnostic tool in MPM management. MiR-20a and miR-548a-3p were assessed in sera of healthy controls, asbestos-exposed and MPM patients using qRT-PCR. Their expression was positive in 91.6 and 96.7% MPM cases respectively, with a 100% of sensitivity as diagnostic MPM biomarker when used in combination (68).

Diagnostic approaches very different from those described above in this section have been used in some other works (69–72).

The epigenetic silencing of miR-34b/c plays a crucial role in the pathogenesis of MPM and in about 90% of MPM cases miR-34b/c is downregulated by DNA methylation (39). Using a digital methylation specific PCR assay, Muraoka et al. analyzed serum samples of 41 healthy controls, 21 asbestos-exposed and 48 MPM patients and demonstrated that a high degree of methylation of miR-34b/c in serum-circulating DNA is associated with MPM diagnosis and with higher MPM stage in patients with previous MPM diagnosis (72).

Cavalleri et al., using an OpenArray method, investigated the expression of 754 miRNAs in the plasmatic extracellular vesicles of 19 asbestos-exposed and 23 MPM patients, and found 55 differentially expressed miRNAs. Among these, 16 were confirmed by RT-qPCR in the validation phase. MiR-30e-3p, miR-98, miR-103a-3p, miR-148b, and miR-744 were the best discriminating miRNAs, and the combination of miR-30e-3p and miR-103a-3p was reported as the most discriminating one with a specificity of 80.0% and a sensitivity of 95.5%. This study is the only one using miRNA quantification in plasma exosomes. This new diagnostic approach is very interesting because it may provide a huge number of information about miRNA release mechanisms but it has the disadvantage of being very expensive (69).

The role of miR-103 family as diagnostic biomarker has been previously shown also by Weber et al. In two consecutive works, the authors used a totally different diagnostic technique based on the identification and quantification of miRNAs in the cellular fraction of human peripheral blood (70, 71). In the first pilot study, published on 2012, the investigators enrolled 17 asbestos-exposed and 23 MPM patients. Analyzing a panel of 328 miRNAs with microarrays, they found the low expression of miR-20a and miR-103. Quantitative-RT-PCR was used for validation phase in 25 healthy subjects, 17 asbestos-exposed and 23 MPM patients and confirmed only miR-103 as significantly down-expressed in the cellular fraction of MPM patients' blood. The authors calculated a specificity of 76% with a sensitivity of 78%, and a specificity of 71% with a sensitivity of 83% for the discrimination of MPM from healthy and asbestos-exposed subjects, respectively (70).

In the second study, the authors evaluated the performance of the combination of miR-103a-3p and mesothelin quantification as diagnostic biomarker in MPM. The analysis was performed on 52 asbestos-exposed and 43 MPM male patients. Mesothelin concentration was determined in plasma samples using ELISA test whilst the levels of miR-103a-3p was assessed in the blood cellular fraction using RT-qPCR. For the discrimination between asbestos-exposed and MPM patients miR-103a-3p, mesothelin and the combination of both showed 89, 74, 95% and 63, 85, 81% of sensitivity and specificity, respectively (71).

## Potential Therapeutic Role

Since their discovery, miRNAs have always been considered as one of the most interesting therapeutic prospects for cancer treatment. Their ability to target multiple cell pathways and the important regulatory role they play in almost all the mechanisms underlying cell replication and tumor progression, have made scientists to believe that they could be widely exploited to increase anti-tumor response and to reduce drug resistance. Considering that a huge number of miRNAs are down-regulated in many cancer types, the great part of the tested therapeutic strategies are based on the possibility to restoring the miRNAs function, often through the delivery of the down-expressed miRNAs inside the tumor cell. In MPM this new therapeutic approach has been experimented in cell lines and mouse models. Various miRNAs (let-7a, miR-16, miR-34b-c, miR-126, mir-145, miR-193a-3p) and various delivery systems have been tested obtaining interesting results in terms of tumor growth inhibition (29, 31, 33, 42–44, 48, 49).

However, despite these interesting premises, to date the results of only one clinical trial (NCT02369198) are available in human subjects. In this phase I, open-label, dose-escalation study, the authors aimed to investigate the safety, the optimal dose and the activity of TargomiRs in MPM patients. TargomiRs are minicells (EnGeneIC Dream Vectors) loaded with miR-16 mimic and targeted against EGFR. The drug was designed with the aim to restore the frequent down-expression of miR-16 in MPM. Twenty-seven patients (with diagnosis of EGFR positive MPM progressed after chemotherapy) were enrolled between September 2014 and November 2016 (26 patients were treated). The investigators found that 5 × 10^9^ intravenous TargomiRs once weekly was the maximum tolerated dose. The most common adverse events were transient lymphopenia (96%), hypophosphataemia (65%), and increased transaminase levels (23%). Cardiac events (18%) occurred in five cases including one case of ischaemia and one case of Takotsubo cardiomyopathy. The drug showed early signs of activity. The median OS was 200 days (95% CI 94-358) with 5% of partial response, 68% of stable disease, and 27% of progressive disease registered as best response (80). The toxicity profile and the initial activity signs, make the development of this drug interesting, especially in association with chemotherapy and or immunotherapy.

Sayeed et al. investigated the potential role of dietary phytochemicals as possible preventive and/or therapeutic tool in MPM in a very interesting review (81). Nevertheless, at the present, only one dietary phytochemical (ursolic acid) (30) has been shown to have miRNA regulatory activity in MPM. The research in this field is at a very preliminary level and a completed opinion cannot be expressed yet.

## Conclusions

Our review strongly supports the idea that the detection, quantization and analysis of miRNAs in MPM tissues and biological fluid samples have a great potential both from diagnostic and therapeutic point of view. Nevertheless, the literature analysis showed multiple limitations in the discussed studies.

One potential explanation for the strong diversity of data obtained from different studies is the heterogeneity in the quantification methods and in the type of samples and controls used. In particular, the adoption of various technical approaches among the analyzed studies, based on extremely different chemistries, represent a limitation for the identification of miRNA candidates with a consistent differential expression in diverse analyzed populations. It is desirable that the use of RNA high throughput sequencing systems will provide more reliable and reproducible data in future investigations, with a more clear clinical application.

Furthermore, in some studies data are poorly defined and some important information are not provided and/or the statistical analysis is not adequate. In order to improve these weaknesses, it is critical that future studies will use more uniform controls for their quantitative evaluations. In particular, while some studies involved normal healthy patients, others included in the same control population patients affected from non-cancer pulmonary disease or asbestos-exposed non-cancer patients. This last approach might lead to misidentification (or lack of identification) of microRNAs whose expression is also altered in these pathological statuses. For these reasons, there is limited reproducibility in the available results, which strongly affects the possibility of meta-analysis of published studies.

Finally, future studies would strongly benefit from the inclusion of additional clinic/pathological parameters of the included patients, such as histotype, disease status, and treatments. Stratification of patients based on these additional parameters might allow a better characterization and classification of MPMs.

However, despite these critical points, several miRNAs and/or miRNA families able to modulate crucial cell functions such as methylation, autophagy, apoptosis, proliferation, invasion, migration, and chemo/radio-resistance have been recently discovered and their knowledge has been deepened also in MPM. The role of various miRNAs as diagnostic or prognostic biomarkers in MPM has been confirmed in more than one study and in some cases it is becoming more and more solid (miR-16, miR-103, miR-126, miR-145, and miR-200c), and could pave the way to their clinical testing, both as diagnostic/prognostic markers, and as therapeutic agents.

Initial data on the use of miRNA replacement therapy in humans are starting to be published and the preliminary results in MPM patients are encouraging (82, 83). However, these therapeutic approaches are still lacking appropriate clinical validation, and several issues will have to be solved before they could be considered for the clinical practice. In particular, it is still unclear how miRNA-based therapies, using mimics or inhibitors, could affect the physiology of normal cells, and there is a still-unmet need of cancer-specific delivery systems that could limit undesired effects of these treatments.

In conclusion, this literature review, by highlighting the extreme heterogeneity of studies analyzing the role of microRNAs in MPM, wants to urge a coordinated collaboration between the main international groups working in this research field. In fact, the future scenario of MPM patients will see relevant clinical improvements only through coordinated efforts of multiple basic and translational research studies.

## Author Contributions

GL, AT, and DP participated in the designing of the manuscript and draft it. MC, GG, FdB, and MG participated in drafting the manuscript and critically revised it. GL, AT, and DP designed and coordinated the manuscript. All the authors read and approved the final manuscript.

### Conflict of Interest Statement

The authors declare that the research was conducted in the absence of any commercial or financial relationships that could be construed as a potential conflict of interest.
